# Protective Behaviors of Bio-Inspired Honeycomb Column Thin-Walled Structure against RC Slab under Impact Loading

**DOI:** 10.3390/biomimetics8010073

**Published:** 2023-02-09

**Authors:** Shijie Wang, Hongxiang Xia

**Affiliations:** 1 College of Civil and Architectural Engineering, Heilongjiang Institute of Technology, Harbin 150050, China; 2 School of Civil Engineering, Northeast Forestry University, Harbin 150040, China

**Keywords:** RC slab, honeycomb structure, buffer interlayer, structural impact, energy absorption

## Abstract

In order to protect the reinforced concrete (RC) slab structure from damage under some accidental conditions, such as impacting and explosion, we used bio-inspired honeycomb column thin-walled structure (BHTS) to serve as a buffer interlayer for the concrete structure inspired by the biological structure of beetle’s elytra. The mechanical properties of AlSi10Mg used to fabricate the BHTS buffer interlayer were determined by low- and medium-speed uniaxial compression tests and numerical simulations. Subsequently, based on the drop weight impact test models, the effect of the buffer interlayer on the response of the RC slab under the drop weight tests with different energy input was compared by the impact force and duration, maximum displacement and residual displacement, energy absorption (EA), energy proportion, and other indicators. The results show that the proposed BHTS buffer interlayer has a very significant protection effect on the RC slab under the impact of the drop hammer. Due to its superior performance, the proposed BHTS buffer interlayer provides a promising solution for EA of augmented cellular structures widely used in defensive structural components, such as floor slabs, building walls, etc.

## 1. Introduction

RC has been widely used in various of defense and civil engineering fields, such as bridges, dams, and nuclear power plant structures, to resist various loads [1,2,3]. RC slabs are the commonly used structural members in buildings, yet impact loads are often overlooked in the design of slabs [4]. Therefore, it is important to understand the failure characteristics of RC slabs under such impact loads [5,6]. Work in this area continues to be driven by a wide range of applications, such as RC structures designed to withstand unexpected load scenarios, such as rockfall impacts [7,8]; collisions of vehicles or ships with buildings, bridges, or offshore installations [9,10]; and structures for high-threat or high-risk applications, such as military defense structures or nuclear facilities [4,10]. Therefore, many scholars have done a great deal of work in developing impact-resistant design procedures and improving the performance of RC structures withstand impact loads [11,12,13,14]. However, the number of studies under dynamic loading is very limited compared to the studies under static loading. The main reason for the insufficient research is that the analysis and design of structures subjected to dynamic shock loads are often very complex, and these analyses become more complex when RC is used as a nonlinear material [15,16].

Many countries are constantly developing many protective structures designed to reduce impact damage [17,18,19,20,21]. Nowadays, many scholars have adopted similar methods, such as increasing the reinforcement ratio and composite reinforcement to strengthen the RC slab to improve the impact stiffness of the structure [4,22,23], but the concrete parts directly bear the impact load by this method, which will still cause great damage to the concrete structure. The ideal protective effect is to sacrifice the replaceable protective member, thus avoiding greater damage to the concrete main structure, which requires the protective member to have excellent EA capacity under compressive load. Pham et al. [24], Sukontasukku et al. [25], and Sun et al. [26] noticed that buffer materials applied to RC structures can delay the impact time, reduce impact forces, damage, and the acceleration of structures in impact tests. Studies by Hong Hao et al. [27,28] have showed that the use of different interlayer in the impact zone results in different impact loading rates and shock pulses acting on the beam. Harder contact conditions lead to higher loading rates, higher peak impulses, and shorter durations. In contrast, soft contact reduces loading rates and impact forces and extends pulse duration. Lower loading rates reduce the inertial effects of concrete structures, making them more susceptible to bend-controlled failure patterns.

**Figure 1:**
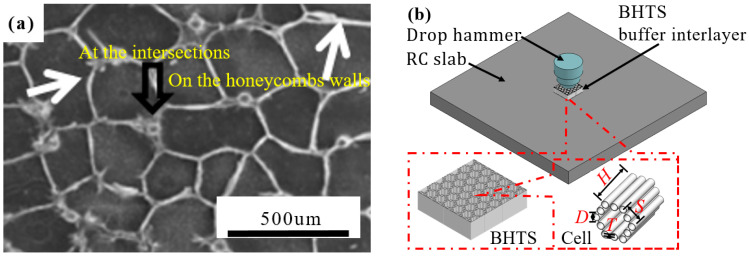
Bionic honeycomb structure and application: (**a**) micromorphology of honeycomb structure with columns [41]; (**b**) schematic diagram of the drop weight test.

Over the years, honeycomb structures (HS) have been found to be effective in improving EA capacity, are widely used in the field of impact resistance [29] and are a common method in practical projects to achieve the goal of lightening the buffer interlayer [30,31,32]. As humans deepen their exploration of nature, HS also draw many inspirations from animals and plants [33,34,35,36,37]. Ngoc San Ha et al. designed a bio-inspired hierarchical circular honeycomb (BHCH) based on the stratified structure of wood [33]. Studies have shown that the relative stiffness, intensity, and EA of BHCH are obviously higher than that of other common cellular structures. Xiang et al. [38] investigated the mechanical properties of beetle’s internal structure under axial impact loading studied by Chen et al. [36,39,40,41,42,43] (Figure 1a), namely, bio-inspired honeycomb column thin-walled structure. Hao [44] is also based on Chen et al. Numerical simulations of BHTS were performed. The research results showed that BHTS has better EA characteristics under axial load than ordinary HS, and this bionic structure is expected to be used as a replaceable device for automobiles to improve collision resistance and lightweight level. The above studies illustrate the potential of BHTS as excellent EA devices.

In recent years, additive manufacturing technology has developed rapidly with high manufacturing accuracy and high material utilization [45,46]. Ngoc San Ha et al. studied the proposed structure EA properties by mimicking the wood’s layered structure through AM technology [33], providing a good solution to the problem of manufacturing complex HS. Xiang et al. [38] and Hao [44] et al. designed some aluminum alloy BHTS structures with excellent absorption capacity.

The motivation and research gap of this paper are as follows:

- BHTS needs to be studied and applied in more depth: the scholars above only made preliminary studies on the performance of BHTS structures and underestimated the strain rate effect of aluminum alloy materials under impact load, and neglected the material failure of aluminum alloy tubes, and did not further study the design structure in actual engineering applications.
- Based on previous research [47,48,49], this paper further studies the proposed BHTS, and intends to apply AM technology to the integrated and efficient production of BHTS, so as to achieve mass production in practical projects.
- The performance of this lightweight buffer interlayer in RC slabs (Figure 1b) was studied by dropping weight impact model to provide a reliable reference basis for large-scale AM of the BHTS.

## 2. Materials and Methods

### 2.1. Design of BHTS

In this study, we proceeded from the internal structure of the beetle elytra. According to the design concept of Xiang and Hao proposed [38,44] and the Chinese code [50], we adjusted the geometric parameters of the BHTS-3 type model designed by Hao [44], and improved the design of the BHTS to better meet the scope of AM, as shown in Figure 1b: The side length *S* (10 mm), the wall thickness *T* (0.3 mm) and the height *H* (30 mm) of the structure are fixed and the conventional honeycomb. The BHTS is equipped with hollow columns at the center of the honeycomb wall and the wall connection with the circular tube diameter *D* of 4 mm. 

### 2.2. Test Methods

#### 2.2.1. Low Strain Rate Uniaxial Compression Test

**Figure 2:**
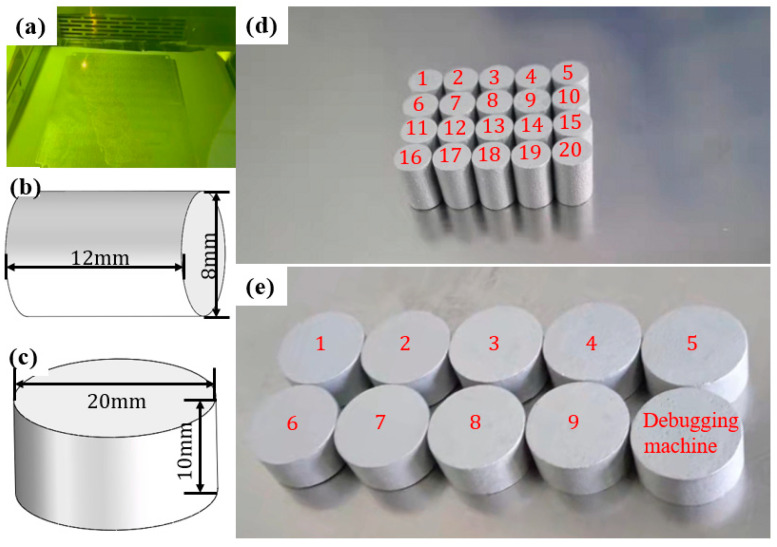
Specimen manufacturing and dimensions: (**a**) AM process; (**b**,**c**) specimen dimensions; (**d**,**e**) specimens manufactured.

**Figure 3:**
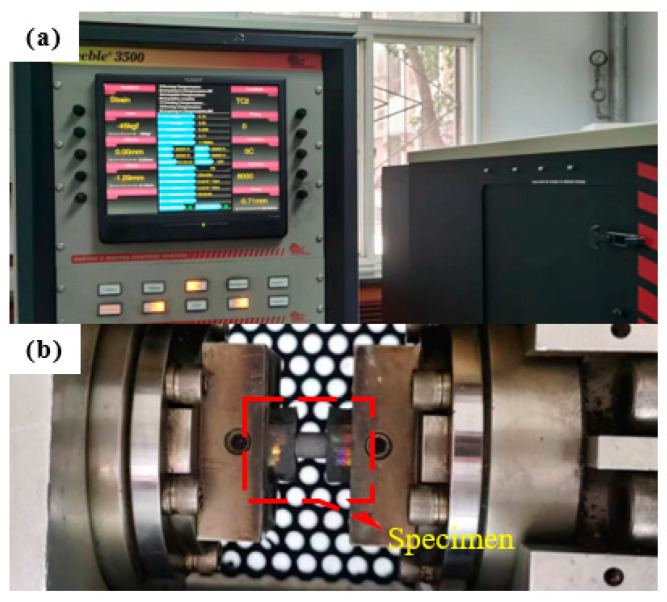
Loading process of specimen: (**a**) control system; (**b**) specimen loading.

To determine the mechanical properties of manufacturing the buffered interlayer material AlSi10Mg, reference is made according to Ngoc San Ha, Thong M. Pham, and many other scholars experimenting with the mechanics of 3D printed porous structures [33]: Through the uniaxial tensile test, the constitutive relation of the fabricated material was determined, and the obtained test data was then used to conduct finite element simulation. The results showed that the numerical model can effectively reflect the EA mechanism. According to the experimental philosophy of the above scholars, cylindrical specimens (Figure 2b) with a height of 12 mm, and a diameter of 8 mm were designed. A low-speed uniaxial compression test (Figure 3) was conducted at room temperature (18 °C) using the thermal simulation test machine GLEBLE 3500 (maximum loading speed 1000 mm/s, minimum loading speed 0.01 mm/s) on four sets of 20 AlSi10Mg cylindrical specimens (Figure 2d) made with selective laser melting technique at strain rates of 0.001 s^−1^, 0.01 s^−1^, 0.1 s^−1^ and 1 s^−1^, respectively. The corresponding displacement rates were 1.2 × 10^−2^ mm/s, 1.2 × 10^−1^ mm/s, 1.2 mm/s and 12 mm/s, respectively, to obtain the model data of the entire quasi-static compression process. Each sample was cleaned with ethanol to remove dust and other potential contamination from its surface before loading and record vertical displacement and applied load. The measured Young’s modulus of AlSi10Mg is 69 ± 5 GPa, the initial yield strength is 220 ± 15 MPa, and the Poisson’s ratio is 0.3.

#### 2.2.2. Medium Strain Rate Uniaxial Compression Test

**Figure 4:**
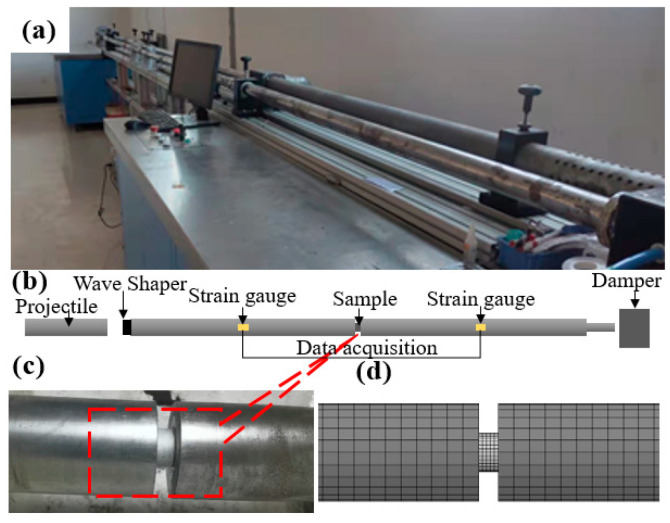
SHPB impact tests: (**a**) overview of the SHPB system in the laboratory; (**b**) schematic of the SHPB testing system; (**c**) AlSi10Mg specimen between the incident and transmission bars; (**d**) finite element model.

Three sets of nine AlSi10Mg cylindrical samples (Figure 2c,e) were subjected to the Split Hopkinson Pressure Bar (SHPB) uniaxial compression test (Figure 4a–d) with strain rates of 300 s^−1^, 800 s^−1^, and 1300 s^−1^, respectively, to obtain dynamic model data for their entire compression process. The diameter of the incident and transmission bars is 50 mm, and the length is 2.5 m. The strain slab is in the middle of the incident and transmission bars (Figure 4b), and the end of the transmission bar is a damping cylinder. The cylindrical specimen is designed to be 10 mm in height, and 20 mm in diameter (Figure 2c). The two-wave formula has been used in many experiments [51,52]. The stress, strain, and strain rate in the specimen can be expressed by two sets of strain signals in the equations, respectively:

(1) $$\sigma_{s}\left(t\right)=E\frac{A}{A_{s}}\varepsilon_{t}\left(t\right)$$

(2) $$\varepsilon_{s}\left(t\right)=\frac{2C_{0}}{L_{s}}\int_{0}^{t}\varepsilon_{r}\left(t\right)dt$$

(3) $${\dot{\varepsilon }}_{s}\left(t\right)=\frac{2C_{0}}{L_{s}}\varepsilon_{r}\left(t\right)$$

In Equations (1)–(3), A, E, $C_{0}$, $S_{s}$, and $L_{s}$ are the cross-sectional area of the bar, the modulus of elasticity, the wave velocity of the elastic compression wave, the cross-sectional area of the specimen, and the length of the specimen; and $\varepsilon_{i}\;\left(t\right)$, $\varepsilon_{r}\;\left(t\right)$, $\varepsilon_{t}\;\left(t\right)$ represent the incident, reflected and transmitted strain pulses of the specimen, respectively.

The SHPB finite element model of AlSi10Mg cylindrical specimen (Figure 4d) was established, and the Cowper-Symonds model was selected [53,54]. Considering the stress hardening effect and the strain rate strengthening effect, the loading process is moderate speed loading with little warming. Therefore, regardless of the softening effect of the resulting temperature on the material, the flow stress expression is as follows:

(4) $$\sigma_{Y}=\left[1+{\left(\frac{\dot{\varepsilon }}{C}\right)}^{\frac{1}{P}}\right]\left(\sigma_{0}+\beta E_{P}\varepsilon_{P}^{eff}\right)$$

**Figure 5:**
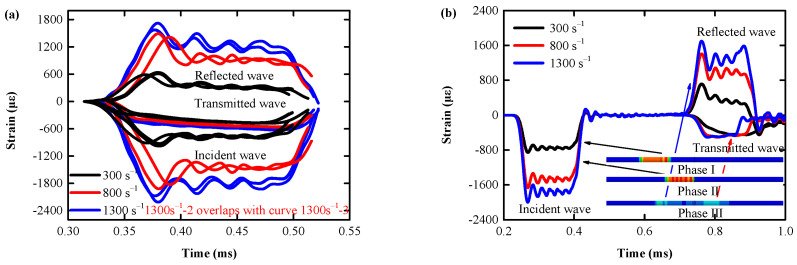
Time travel curves under 300 s^−1^, 500 s^−1^, and 800 s^−1^ strain rate loading: (**a**) test curve; (**b**) FEM curve.

In Equation (4), $\dot{\varepsilon }$ is the loading strain rate C, and P is the strain rate constant. $\sigma_{0}$, β, $E_{P}$, and $\varepsilon_{P}^{eff}$ are the initial yield stress, hardening parameters, plastic hardening modulus and effective plastic strain, which determined according to the test. Waveform design of impact rod, and strikes the same as the actual situation, with a rectangular wave with a period of 0.2 ms (Figure 5b). From Figure 5a,b, it can be seen that the numerical simulation results and the test results have achieved a very close fit (the average peak error of the three types of waves is 7.54%), indicating that the numerical model of AlSi10Mg material has achieved a good fit with the actual mechanical characteristics and can effectively simulate the actual mechanical behavior.

### 2.3. Mechanical Properties of AlSi10Mg

**Figure 6:**
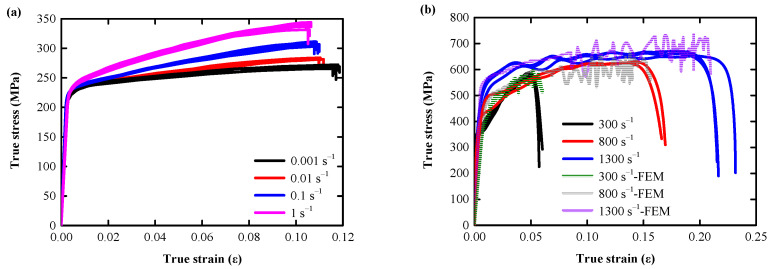
Static/dynamic true stress–strain curve: (**a**) under 0.001 s^−1^, 0.01 s^−1^, 0.1 s^−1^, and 1 s^−1^ strain rate loading; (**b**) under 300 s^−1^, 500 s^−1^, and 800 s^−1^ strain rate loading.

Figure 6a shows the true stress–strain curve of 20 AlSi10Mg cylinders obtained by quasi-static compression. Despite some deviations, these curves consist mainly of three different deformation stages: the linear and nonlinear elastic deformation stages (ε = 0–0.003), the plastic hardening stage (ε = 0.003–0.118), and the failure stage (ε > 0.118): the initial linear cross section of the elastic deformation stage is very short, the AlSi10Mg skeleton begins to bear stress at the beginning of compression and rapidly transitions to the next nonlinear elastic deformation stage; the plastic hardening stage, where the skeleton stress exceeds the elastic limit, resulting in AlSi10Mg yielding, and the curve undergoes an extended platform zone with a large increase in strain; the failure stage, where after reaching the peak stress, the stress decreases rapidly until the specimen is fractured. At the end of this phase, AlSi10Mg basically lost its EA capacity.

Figure 6b shows the true stress–strain curve of 8 AlSi10Mg cylinders obtained by the medium strain rate SHPB compression test (800 s^−1^ strain rate loading invalid for the first data acquisition). The test curve is essentially the same as the quasi-static compression deformation trend, except that the yield strength and the ultimate strength of AlSi10Mg both increase significantly with the strain rate (yield strength/ultimate strength at 300 s^−1^, 800 s^−1^, and 1300 s^−1^ increased by 60.44%/106.69%, 111.11%/118.66%, and 148.12%/134.15%, respectively, compared with quasi-static 0.001 s^−1^ strain rate loading), indicating that AlSi10Mg is a strain rate sensitive material.

## 3. Numerical Simulation

### 3.1. Constitutive Model of Materials

#### 3.1.1. Concrete Model

Numerical simulation is a common way for scholars to study concrete [55,56,57,58,59]. In order to fully understand the impact response of RC slabs, many scholars have developed numerous numerical models of RC slabs in LS-DYNA [55], such as *MAT_PSEUDO_TENSOR,*MAT_WINFRITH_COCRETE,*MAT_JOHNSON_HOLMQUIST_CONCRETE,*MAT_CONCRETE_DAMAGE_REL3, and *MAT_CSCM_CONCRETE, etc. Some available material models have been extensively evaluated for extreme dynamic loads [57,58,59]. In this study, the * MAT_CSCM model was selected. The projection shape of the yield surface on the deviatoric plane is described by Willam–Warnke simulation. The position and size of the reinforced cap surface are determined by the history of stress and strain experienced by the material. The model considers the hardening, damage, and rate dependence of the material. At present, it is widely used in the low velocity impact field of RC structures [60,61]. The specific parameters adopted in this model referenced the literature [61,62] are shown in Table 1.

#### 3.1.2. Steel Reinforcement Model

The steel reinforcement is Hughes-Liu beam element with a 2 × 2 Gaussian cross-sectional area. In order to more conveniently input the formula developed by some scholars to simulate the strain rate effect of steel reinforcement calculation formula under the impact load, the segmented linear plastic model *MAT_PIECEWISE_LINEAR_PLASTICITY [53] was selected.

### 3.2. Strain Rate Effect

#### 3.2.1. Concrete

The effect of tensile strength and compressive strength strain rate under uniaxial stress is considered by the CEB equations [63]. The strain rate enhancement factor is calculated as follows:

(5) $$DIF_{ten}={\left(\frac{\dot{\varepsilon }}{{\dot{\varepsilon }}_{0}}\right)}^{1.016d}\;and\;DIF_{comp}={\left(\frac{\dot{\varepsilon }}{{\dot{\varepsilon }}_{0T}}\right)}^{1.026\alpha_{s}}\;\;for\;\left|\dot{\varepsilon }\right|\leq 30\;s^{-1}$$

(6) $$DIF_{ten}=B_{s}{\left(\frac{\dot{\varepsilon }}{{\dot{\varepsilon }}_{0}}\right)}^{1/3}\;and\;DIF_{comp}=\gamma_{s}{\left(\frac{\dot{\varepsilon }}{{\dot{\varepsilon }}_{0}}\right)}^{1/3}\;\;\;\;for\;\left|\dot{\varepsilon }\right|>30\;s^{-1}$$

where $d=1/\left(10+0.6f_{c}^{\prime }\right)$; $\alpha_{s}=1/\left(5+9f_{c}^{\prime }\right)$; $log_{10}^{B_{s}}=7.112d-2.33$; $log_{10}^{\gamma_{s}}=6.156\alpha_{s}-2$; ${\dot{\varepsilon }}_{0}=30\times 10^{-6}\;s^{-1}$; $f_{c}^{\prime }$ is the unconfined compression strength of the concrete in MPa.

In addition, the elastic modulus is defined as the tangent elastic modulus at the starting point of the concrete stress–strain diagram [63].

(7) $$E_{con}=2.15\times 10^{4}{\left[\frac{f_{c}^{\prime }}{10}\right]}^{1/3}\left(\mathrm{MPa}\right)$$

#### 3.2.2. Steel Reinforcement

This material can also define the effect of strain rate effect on steel yield stress. In this paper, the strain rate effect proposed by Malvar and Crawford [64] is used to quantify the strength increment of steel under dynamic loading conditions. The strain rate relationship between the tensile and compressive dynamic increase factor (DIF) of steel is defined by the following equations [65,66]:

(8) $$DIF={\left(\frac{\dot{\varepsilon }}{10^{-4}}\right)}^{\alpha_{1}}$$

(9) $$\alpha_{1}=0.074-\frac{0.04f_{y}}{414}$$

where $f_{y}$ is the yield strength of the steel. It is noteworthy that in this study, DIF of steel material was prevented from being overestimated at very high strain rates when the strain rate was above 160 s^−1^ [65]. 

### 3.3. Application of Pretightening Force to Concrete Slabs

The expansion is induced by the temperature in the slab roof constraint, and the prestress is applied to the concrete component [67]. The slab roof constraint expands as the temperature rises and, therefore, creates a compressive stress on the concrete. The temperature-induced strain $\varepsilon_{T}$ can be determined by the following equation:

(10) $$\varepsilon_{T}=\Delta T\alpha_{2}=\varepsilon_{c}+\varepsilon_{s}$$

where $\alpha_{2}$ is the thermal expansion coefficient of the slab roof constraint (i.e., $\alpha_{2}=1\times 10^{-5}/{}^{\circ}C$), ΔT is the temperature change of the reference temperature or the initial temperature, and $\varepsilon_{T}$ is the slab roof constraint strain without any contact force limit when the temperature drops. $\varepsilon_{c}$ is the concrete strain, and $\varepsilon_{s}$ is the roof constraint of slab. A constrained pretightening force f is applied to each top slab, which ΔT can be obtained by the following equation [65,67]:

(11) $$\Delta T=\frac{f}{\alpha_{2}E_{S}A_{S}}\left(1+\frac{E_{S}A_{S}}{E_{c}A_{c}}\right)$$

where $\;E_{s}$ and $A_{s}$ are the elastic modulus and cross-sectional area of the slab roof constraint, respectively, $\;E_{c}\;$ and $\;A_{c}\;$ represent the corresponding measured values of the concrete. Thermal *MAT_ADD_THERMAL_EXPANSION is used to define the temperature-related material properties of the support.

### 3.4. Erosion Model for Concrete and Steel Reinforcement Elements

According to the damage of concrete material, erosion is considered to avoid grid entanglement in the event of large deformation to eliminate elements not involved in resisting the application of impact loads during RC slab analysis. Where CSCM material is used when the damage exceeds 0.99 and the maximum primary strain exceeds EROSION-1.0. Taking the different values of the erosion coefficient greater than 1.0 into account, a parameter survey was conducted to assess the overall failure [68]. When the maximum primary strain reaches ± 5.0%, it deleted the concrete grids.

The failure criterion for steel reinforcement is based on the failure plastic strain of the erosion elements available in the material model. When the ultimate plastic strain value determined by the tensile test reached, remove the element from the model, and take 0.18 as the plastic strain limit.

### 3.5. Numerical Simulation

**Figure 7:**
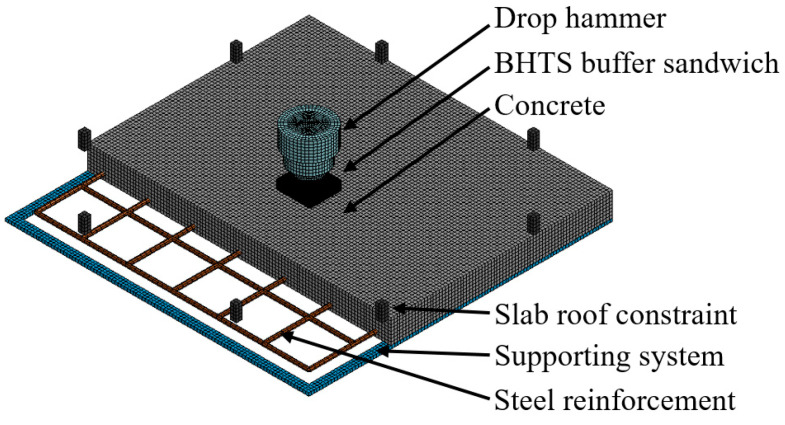
Schematic diagram of FEM model.

The grid size of the concrete slab and support in the three-dimensional x, y, and z are 10 × 10 × 10 mm (Figure 7), considering the bond-slip effect between reinforcement and concrete, a 2-node Hughes-Liu beam unit with a length of 15 mm with 2 × 2 Gauss integral was used to simulate the steel reinforcement. Interfacial adhesion between concrete and steel reinforcement was modeled using the *CONSTRAINED_LAGRANGE_IN_SOLID (CLS) features provided in LS-DYNA [69], see Table 2 for the material parameters.

All translational degrees of freedom of bottom steel plates have been constrained, and the nodes at the bottom of the concrete slab in contact with the steel support adopt the *CONTACT_AUTOMATIC_SURFACE_TO_SURFACE automatic surface contact algorithm to avoid penetration between the concrete and the steel support grids. The contact part between the slab roof constraint and the RC slab, and the contact between the drop hammer and the RC slab, adopt the *CONTACT_ AUTOMATIC_SIGLE_SURFACE_TO_SURFACE automatic single-contact-interface algorithm. Use the keyword *INITIAL _VELOCITY_GENERATION to get the initial speed of the drop hammer. Use the keyword *LOAD_BODY_Z to apply gravity to all node parts of the drop hammer system.

A pre-tightening force of 5 N is applied to each constraint boundary distribution prior to transient analysis in a finite element analysis program. The force and gravity initialization process are accomplished by performing the quasi-static simulation *CONTROL_DYNAMIC_RELAXATION (DR) option within the pseudo time. The *LOAD_THERMAL_LOAD_CURVE is used to define the temperature–time curve for the slab roof constraint. The application process involves defining two temperature–time curves: the first curve maintains the steady-state temperature, and the second curve is the dynamic relaxation curve.

### 3.6. Model Validation

**Figure 8:**
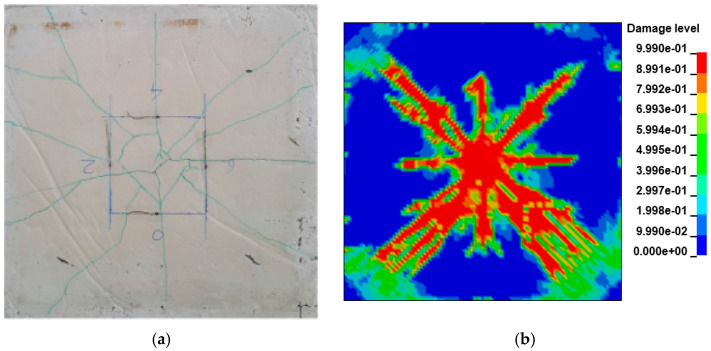
Specimen 1 slab bottom impacted crack distribution: (**a**) experiment [70]; (**b**) numerical simulation.

**Figure 9:**
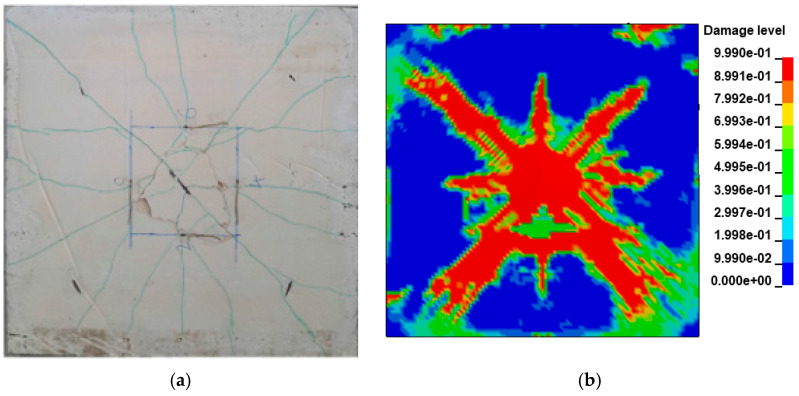
Specimen 2 slab bottom impacted crack distribution: (**a**) experiment [70]; (**b**) numerical simulation.

**Figure 10:**
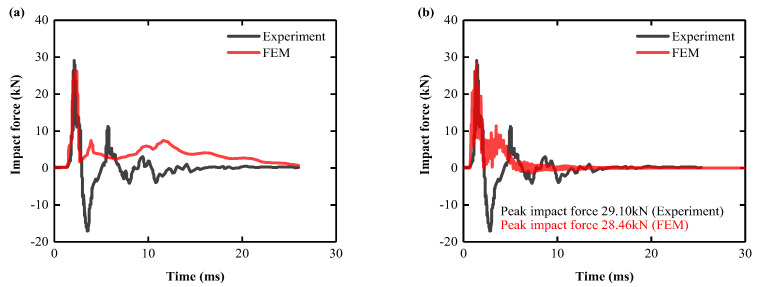
Specimen 1: comparison of impact force between test slab and FEM: (**a**) other scholars [70]; (**b**) authors.

**Figure 11:**
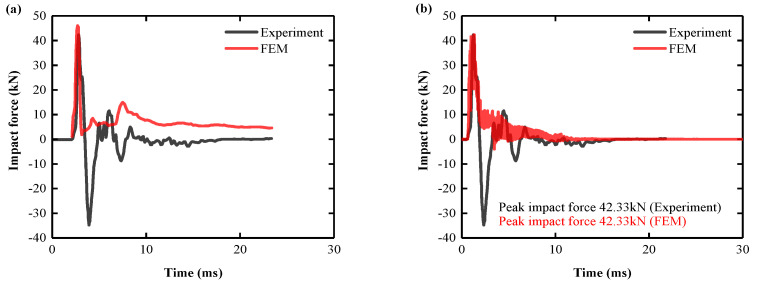
Specimen 2: comparison of impact force between test slab and FEM: (**a**) other scholars [70]; (**b**) authors.

**Figure 12:**
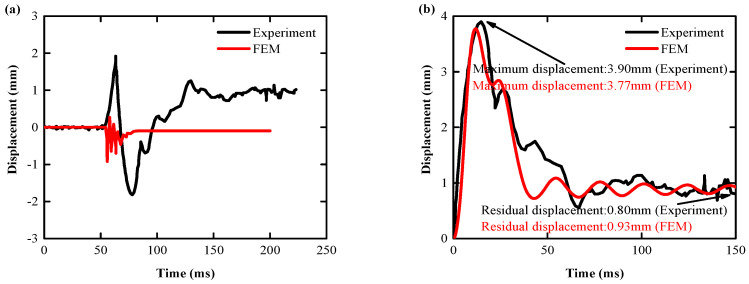
Specimen 1: comparison of test slab and FEM displacement results: (**a**) other scholars [70]; (**b**) authors.

**Figure 13:**
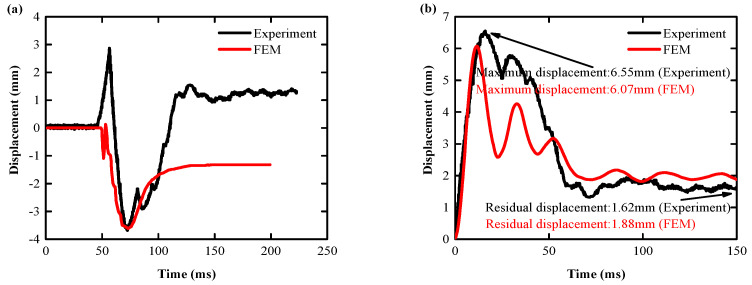
Specimen 2: comparison of test slab and FEM displacement results: (**a**) other scholars [70]; (**b**) authors.

In order to verify the material model and numeric modeling algorithm used, this study conducted numerical simulation of the drop weight impact test of RC slab structure by Selim Sengel et al. [70]. The test results of Specimen 1 and Specimen 2 were compared with the numerical results of FEM for failure phenomena (Figure 8 and Figure 9), the magnitude and duration of peak impact force (Figure 10 and Figure 11), and the peak displacement and residual displacement (Figure 12 and Figure 13).

#### 3.6.1. Damage Status

(1) Specimen 1 crack distribution comparison

The comparison between the experiment and FEM results of the Specimen 1 slab structure at 0.618 kJ energy input is shown in Figure 8a,b, respectively. From the above figure, it can be seen that the crack distribution of the Specimen 1 slab structure is cross-shaped and X-shaped superimposed distribution after the impact of the drop hammer. The severity of crack damage decays from the center to the periphery, with the center being the most damaged by the impact.

(2) Specimen 2 crack distribution comparison

The experiment and numerical comparison of Specimen 2 slab structure at 1.236 kJ energy input are shown in Figure 9a,b, respectively. From the above figure, it can be seen that the Specimen 2 slab structure is similar to Specimen 1. The crack distribution is still cross-shaped and X-shaped superimposed distribution after the impact by the drop hammer. The crack damage degree decays from the center to the surroundings, and the center is the most damaged at the impact site. In the tests, the bottom slab of Specimen 2 exhibited obvious shear cracks under the impact of the drop hammer, which were distributed around the impact location of the drop hammer. Meanwhile, the bottom slab of the Specimen 2 numerical model also showed shear cracks at the impact position around the drop hammer, accompanied by concrete spalling off (Figure 9b).

#### 3.6.2. Impact Force

(1) Specimen 1 impact force comparison 

The impact force–time curve of the experiment of the Specimen 1 slab structure and the numerical simulation of the tester is shown in Figure 10a. The impact force–time curve simulated in this paper is shown in Figure 10b. Since the design of the force transducer in the test adopts the hammer drop rod [70], so that the compression wave propagates from the hammer rod to the rod end (fixed end), and the reflected compression wave is reflected to the free end (hammer end), the direction of the reflected stress wave (tensile wave) is changed, so it is only necessary to compare the peak impact force and duration of the impact force. Comparative analysis results show that the peak force magnitude difference is 2.20% and the impact duration difference is 4.00%, indicating that the numerical simulation results can effectively reflect the impact of the real Specimen 1 slab structure.

(2) Specimen 2 impact force comparison

The impact force–time curve of the experiment of the Specimen 2 slab structure and the numerical simulation of the tester are shown in Figure 11a. The impact force–time curve simulated in this paper is shown in Figure 11b. As with the Specimen 1 slab structure, the peak impact force and duration of impact force are compared. Comparative analysis results showed that the peak force size matched perfectly, and the difference in impact duration was 2.24%, indicating that the numerical simulation results effectively reflected the impact of the real Specimen 2 slab structure.

#### 3.6.3. Displacement Response

(1) Specimen 1 displacement response comparison

The displacement-time curve between the test of the Specimen 1 slab structure and the numerical simulation of the tester are shown in Figure 12a. The displacement-time curve simulated in this paper is shown in Figure 12b. Two key indicators [11] were compared over a relative time horizon of 0–100 ms: peak displacement, and residual displacement. Comparative analysis results showed that the peak displacement difference was 3.33%, and the residual displacement difference was 16.25%, indicating that the numerical simulation results reflected the impact of the real Specimen 1 slab structure well.

(2) Specimen 2 displacement response comparison

The displacement-time curve between the test of the Specimen 2 slab structure and the numerical simulation of the tester is shown in Figure 13a. The displacement-time curve simulated in this paper is shown in Figure 13b, and the peak and residual displacements within the relative time range of 0–150 ms are compared with the test data. Comparative analysis results showed that the peak displacement difference was 7.33%, and the residual displacement difference was 16.05%, indicating that the numerical simulation results better reflected the impact of the real Specimen 2 slab structure.

According to the above verification results, the comparison results of failure phenomenon, impact force and displacement match the experiment data well, and the dynamic response of the test slab under the impact load can be well simulated. Therefore, it can be considered that the current numerical model, including the adopted material model, and the corresponding numerical algorithm can analyze the dynamic behavior of the bidirectional simply supported slab under the action of vertical impact load.

## 4. Results and Discussion

### 4.1. Impact Crack Patterns

**Figure 14:**
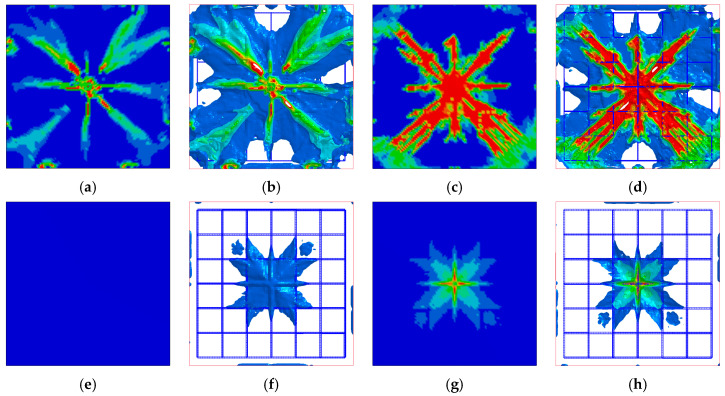
Plastic deformation of RC slab under 0.618 kJ energy input: (**a**,**b**) RC slab top; (**c**,**d**) RC slab bottom; (**e**,**f**) BHTS-RC slab top; (**g**,**h**) BHTS-RC slab bottom.

**Figure 15:**
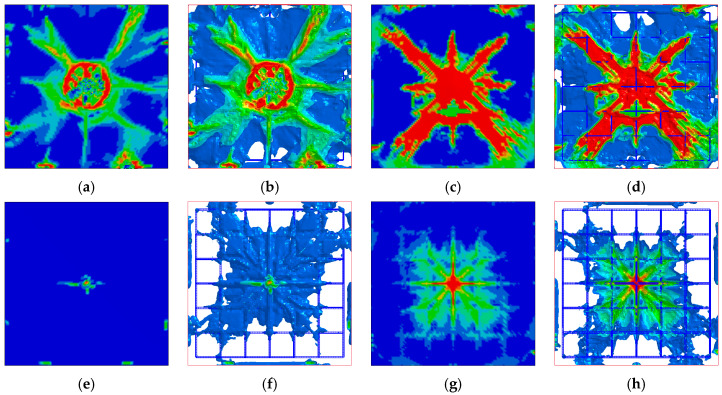
Plastic deformation of RC slab under 1.236 kJ energy input: (**a**,**b**) RC slab top; (**c**,**d**) RC slab bottom; (**e**,**f**) BHTS-RC slab top; (**g**,**h**) BHTS-RC slab bottom.

**Figure 16:**
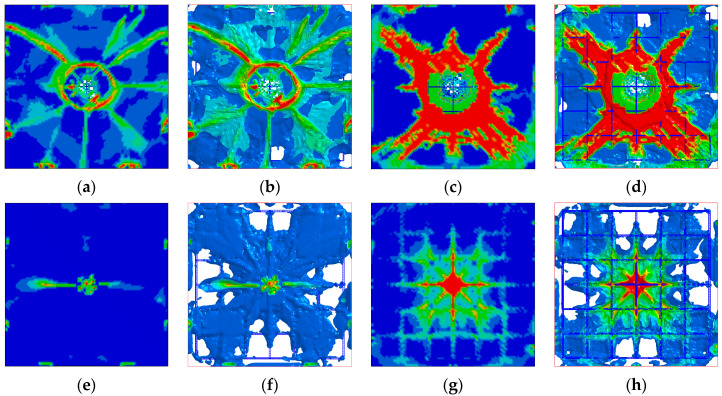
Plastic deformation of RC slab under 1.854 kJ energy input: (**a**,**b**) RC slab top; (**c**,**d**) RC slab bottom; (**e**,**f**) BHTS-RC slab top; (**g**,**h**) BHTS-RC slab bottom.

The length *L*, width *W*, height *T*, side length *s*, thickness *t,* and cylinder diameter *D* of the designed BHTS buffer interlayer structure are, respectively fixed at 110 mm, 104 mm, 30 mm, 10 mm, 0.3 mm, and 4 mm, and the design model is shown in Figure 1b. Place the BHTS buffer interlayer between the drop hammer and the RC slab, referring to this combination structure as the BHTS-RC slab. Drop weight impact test at 0.618 kJ, 1.236 kJ, and 1.854 kJ, respectively, energy input was performed on the RC slab. Figure 14, Figure 15 and Figure 16 shows the numerical calculation cloud chart of the damage status. The darker the color, the greater the plastic deformation of the part and the more serious the damage are. It can be seen from the figure that the RC slab has obvious deformation characteristics under the 0.618 kJ energy input. Among them, the RC slab has more obvious plastic deformation at the impact and shear position, and the fracture trend of the top and bottom surfaces is cross-shaped and X-shaped expansion in the direction of the support along the impact position (Figure 14a–d). The BHTS buffer interlayer protects the top slab directly under the impact load at an energy input of 0.618 kJ. No obvious plastic deformation on roof slab. The bottom slab is only slightly plastically deformed. The crack trend is cross-shaped and expands from the impact center to the surroundings (Figure 14 e–g). The deformation characteristics of the top and bottom the RC slab under the energy input of 1.236 kJ are more obvious than the former (0.618 kJ). The crack distribution trend is also cross-shaped and X-shaped. The top slab concrete is crushed in the collision center (Figure 15a,b), and the concrete cover of the bottom slab is falling off (Figure 15c,d). The BHTS buffer interlayer, under the energy input of 1.236 kJ pulse, caused only slight plastic deformation of the top and bottom slabs (Figure 15e–g), and the crack trend showed a cross-shaped distribution that diverged from the center of impact to the surroundings. Under the energy input of 1.854 kJ, the RC slab underwent obvious plastic deformation, and the top and bottom slabs were damaged to different degrees: The surface of the RC top slab was penetrated with the bottom slab concrete was spalled off, the internal reinforcing structure was exposed, and the scabbing mode (Figure 16a–d) appeared [12,71,72]. The impact center of the top slab and the bottom slab concrete were annularly damaged, where the top slab concrete was mainly crushed and shear damaged, the bottom slab concrete was mainly tensile fracture, and the cracking trend of the top slab and the bottom slab concrete was still cross-shaped and X-shaped overlapping mode. The RC slab structure protected with BHTS buffer interlayer remained intact under 1.854 kJ energy input, and the top and bottom slabs were not damaged, resulting in only slight plastic deformation (Figure 16e–g). From the above test model results, it can be seen that the structural failure of the RC slab protected by the BHTS buffer interlayer is significantly slower than that of the RC slab.

### 4.2. Impact Force Response

**Figure 17:**
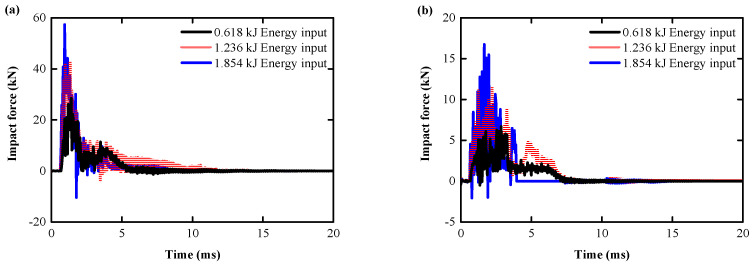
Impact-resistance analysis through the tested peak impact force: (**a**) RC slabs; (**b**) BHTS-RC slabs.

The impact force–time curves are shown in Figure 17a,b. The results show that all the impact specimens exhibit similar fluctuation trend under the impact load. The RC and BHTS-RC slabs impact force increases with the increase in the pulse excitation intensity, and the BHTS-RC slab’s impact duration increased compare with the RC slab’s.

**Figure 18:**
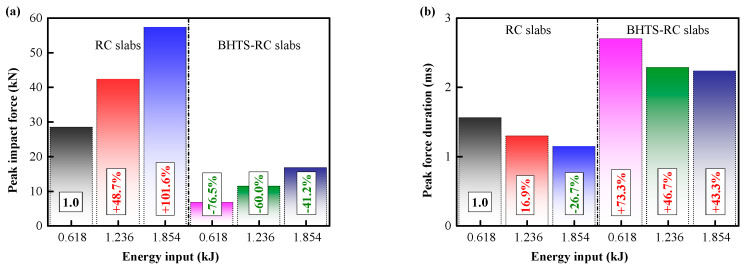
Impact characteristics: (**a**) peak impact force; (**b**) peak force duration.

In order to visually represent the degree of impact deceleration of the RC slab by the BHTS buffer interlayer, the impact force generated by the RC slab under 0.618 kJ pulse excitation is set as the standard value 1. The smaller the ratio to the standard value, indicating that the smaller the impact force, the more beneficial it is for the slab to maintain the integrity of the structure under the impact load. The protective effect of BHTS-RC slabs under all kinds of loading conditions is very obvious, showing that the peak impact force is significantly less than that of ordinary RC slabs, with a minimum and maximum amplitudes of 41.20% and 76.50%, respectively, and an average amplitude of 59.23% (Figure 18a). The peak impact duration of the RC slab under 0.618 kJ pulse excitation is set as the standard value 1, and the ratio with the standard value is greater, which indicates that the more favorable the slab to resist the impact load. Under various loading heights, the peak impact period of the BHTS-RC slab showed a significant increase compared to that of the RC slab, with a minimum and maximum amplitudes of 43.30% and 73.30%, respectively, and an average amplitude of 54.43% (Figure 18b), indicating that the BHTS buffer interlayer effectively reduces the inertial force generated by the effective mass part of the RC slab during the impact [27,28]. 

### 4.3. Displacement Response 

**Figure 19:**
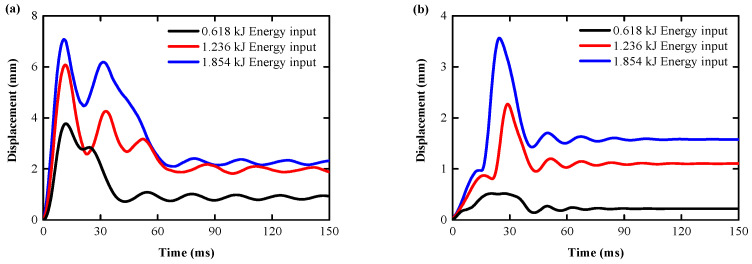
Impact-resistance analysis through the tested displacement: (**a**) RC slabs; (**b**) BHTS-RC slabs.

**Figure 20:**
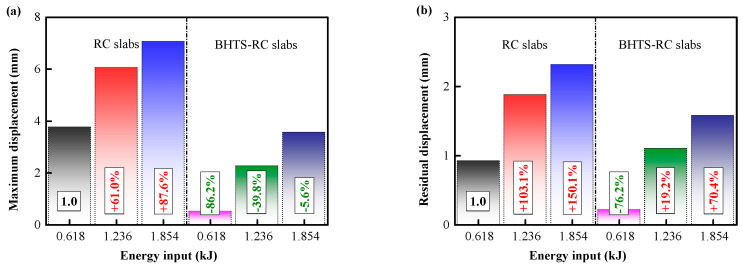
Displacement characteristics under impact load: (**a**) peak displacement; (**b**) residual displacement.

Figure 19a,b shows the displacement curve of the RC slab and the BHTS-RC slab at the 0.618–1.854 kJ energy input. In these slabs, the maximum displacement and residual displacement both increase with the increase in the input energy, and in the RC slabs fitted with BHTS buffer interlayer, the maximum displacement and residual displacement are smaller than the RC slabs. In order to visually indicate the degree of reduction in the displacement of the RC slab by the BHTS buffer interlayer, the maximum displacement and residual displacement caused by the RC slab dropping at 0.618 kJ energy input are, respectively, set as the standard value 1. This indicator reflects the change in the impact stiffness of the RC slab, and the smaller the ratio to the standard value, indicating that it is more favorable for the RC slab to resist the impact load. The research results show that the protective effect of the BHTS buffer interlayer at various energy input condition is very obvious, and the maximum deflection is greatly reduced. The minimum and maximum amplitudes are 5.60% and 86.20%, respectively, and the average amplitude is 43.87% (Figure 20a). The residual displacement trend is the same. The minimum and maximum amplitudes are 19.2% and 76.2%, respectively, and the average amplitude reaches 55.27% (Figure 20b)**.** The impact stiffness of the slab is significantly enhanced by the adoption of BHTS buffer interlayer protection, which indicates that the impact resistance of the RC slab is greatly improved by the BHTS buffer interlayer.

### 4.4. Energy Consumption

**Figure 21:**
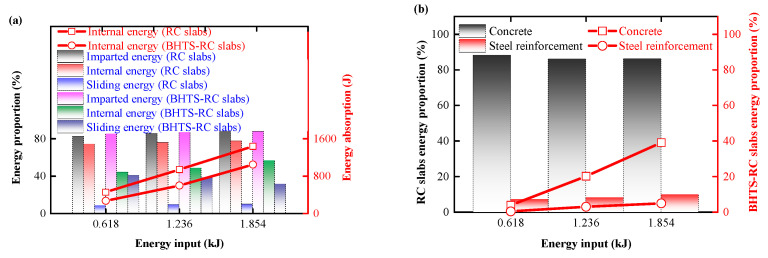
Energy distribution characteristics of RC and BHTS-RC slabs: (**a**) energy transfer efficiency; (**b**) the redistribution of energy within each component of the RC slab caused by the BHTS buffer interlayer.

Compared the distribution of energy absorbed by RC and BHTS-RC slab parts during impact. Apart from sliding energy, the proportion of internal energy in the energy consumption of slab roof restraint and drop hammer is small and can negligible. The change situation of each part of energy was calculated, and the transfer energy after impact stabilization was simplified to the sum of internal energy and sliding energy. It can be found that the proportion of transfer energy and initial kinetic energy of all slabs remained basically unchanged during the transfer process. The average energy transmission efficiency of RC slabs was 85.28%, and the average transmission efficiency of BHTS-RC slabs was 86.63% (Figure 21a), and there was no obvious change in the energy distribution size before and after the reinforcement. At the same time, the internal EA of all slabs increased linearly with the input energy increased, and the average conversion efficiency of the RC slabs was 76.00%, and the average conversion efficiency of the BHTS-RC slabs was 49.74%. Kinetic energy of the difference part (about 27%) was transferred into the sliding energy in the BHTS buffer interlayer, thus causing the conversion efficiency of the two slabs to differ.

Compared the distribution of internal energy of various parts of the slab (concrete, reinforcing steel, BHTS buffer interlayer) during impact. The internal energy consumption is mainly concentrated in the concrete slab, reinforcing steel and BHTS buffer interlayer sections. In order to more clearly express the energy proportion of each part during the impact, the energy distribution within each part is calculated. It can be seen that BHTS caused the energy redistribution in the slab (Figure 21b), and the EA has a significant descent range compared to the RC slab: Under the 0.618 kJ, 1.236 kJ and 1.854 kJ energy input, the proportion of the concrete absorption internal energy in the RC slab is 88.28%, 86.18%, and 86.34%, respectively, and the average proportion is 86.93%, while the proportion of the concrete internal energy in the RC slab protected by BHTS buffer interlayer is 3.89%, 20.19%, and 39.13%, respectively, and the average proportion is 21.06%. The maximum decrease is 84.39% (0.618 kJ), the minimum decrease is 47.21% (1.854 kJ), and the average decrease is 65.86% (total internal energy proportion), and 75.76% (concrete energy proportion). The decrease decreases with the increase in energy input, which is due to the fact that the BHTS buffer interlayer absorbs almost all the input energy when the energy input is low, and almost completely dissipates the input energy when it reaches the RC slab. Figure 14e well illustrates this phenomenon. As the energy input continues to increase, when the input energy is greater than the energy absorbed by the BHTS buffer interlayer under specific conditions, the concrete in the RC slab begins to absorb the energy and develop obvious plastic deformation and destruction phenomenon (Figure 16e). After installing the BHTS buffer interlayer, the average total internal energy proportion of the reinforcing steel decreased by 5.56%, and the average decrease amounted to 66.49% (the ratio of internal energy of the steel reinforcement). The BHTS buffer interlayer greatly reduces the EA of the concrete in the vulnerable part, and also significantly reduces the plastic deformation of the steel reinforcement, indicating that the internal energy redistribution phenomenon produced by the BHTS buffer interlayer during impact is conducive to the slab body to maintain good structural integrity under impact load.

## 5. Conclusions

This paper proposed a comprehensive experimental and numerical analysis to study the energy absorption behavior of the bio-inspired honeycomb column thin-walled structure (BHTS) to mitigate the impact load of RC slab. Based on the analysis above, the following conclusions can be enumerated from the results and discussions:

1. AlSi10Mg is a strain-rate sensitive materials, which has a significant influence on the mechanical behavior of the material and cannot be ignored in numerical simulations;
2. The results of the finite element model verification show that it is in good agreement with the experiment, having a reliable reference value, and can be used for subsequent numerical analysis and expansion research;
3. The BHTS buffer interlayer can cause the redistribution of the absorbed energy of steel reinforcement and concrete in the RC slab: The average total internal energy proportion of steel reinforcement in the RC slab decreased by 5.56%, and the average decrease amplitude was 66.49%; the average total internal energy proportion of concrete decreased by 65.86%, and the average decrease amplitude was 75.76%.

Overall, the research has shown that the proposed BHTS buffer interlayer shows significant energy absorption enhancement as an energy absorber, with great potential in various engineering applications.

## Figures and Tables

**Table 1:** CSCM concrete model properties.

MID	RO	NPLOT	INCRE	IRATE	ERODE	RECOV	ITRETRC
1	2.4 × 10^−9^	1	0	1	1.05	10	0
PRED							
0							
G	K	ALPHA	THETA	LAMDA	BETA	NH	CH
1.125 × 10^4^	1.231 × 10^4^	14.56	0.2979	10.51	1.929 × 10^−2^	0	0
ALPHA1	THETA1	LAMDA1	BETA1	ALPHA2	THETA2	LAMDA2	BETA2
0.7473	1.139 × 10^−3^	0.17	7.014 × 10^−2^	0.66	1.374 × 10^−3^	0.160	0.07014
R	XO	W	D1	D2			
5	90.74	0.05	2.5 × 10^−4^	3.492 × 10^−7^			
B	GFC	D	GFT	GFS	PWRC	PWRT	PMOD
1.00 × 10^2^	9.487	0.1	9.49 × 10^−2^	9.49 × 10^−2^	5	1	0
ETAOC	NC	ETAOT	NT	OVERC	OVERT	SRATE	REPOW
1.003 × 10^−4^	0.78	6.22 × 10^−5^	0.48	21.63	21.63	1	1

When unit = 1, the unit system is MPa, mm, sec, ton/mm^3^, N.

**Table 2:** Specified properties of material models.

Component	Material Model	The Main Parameters of the Material
Concrete	*MAT_CSCM	$\rho =2.4\times 10^{-9}\;t/{\mathrm{mm}}^{3}$ , E=27 GPa, ν=0.2, $f_{c}=20\;\mathrm{MPa}$
Steel reinforcement	*MAT_PIECEWISE_LINEAR_PLASTICITY	$\rho =7.85\times 10^{-9}\;t/{\mathrm{mm}}^{3},\;$ E=200 GPa, ν=0.3, $f_{y}=576\;\mathrm{MPa}$
BHTS buffer interlayer	*MAT_PLASTIC_KINEMATC	$\rho =2.67\times 10^{-9}\;t/{\mathrm{mm}}^{3},\;$ E=70 GPa, ν=0.3, $f_{y}=210\;\mathrm{MPa}$
Drop hammer	*MAT_ELASTIC	$\rho =7.85\times 10^{-9}\;t/{\mathrm{mm}}^{3},\;$ E=200 GPa, ν=0.3
Support	*MAT_ ELASTIC	$\rho =7.85\times 10^{-9}\;t/{\mathrm{mm}}^{3},\;$ E=200 GPa, ν=0.3
Slab roof constraint	*MAT_ADD_THERMAL_EXPANSION	$\alpha_{2}=1\times 10^{-5}$

Note: ρ is the density of material, E is the young’s elastic modulus, ν is the poisson’s ratio, $f_{y}$ is the yield strength, $f_{c}$ is the axial compressive strength of concrete. $\alpha_{2}$ is the thermal expansion coefficient.

## Data Availability

Not applicable.
